# Association of *MC4R* rs17782313 Genotype With Energy Intake and Appetite: A Systematic Review and Meta-analysis

**DOI:** 10.1093/nutrit/nuae075

**Published:** 2024-06-14

**Authors:** Cristina Álvarez-Martín, Francisco Félix Caballero, Rocio de la Iglesia, Elena Alonso-Aperte

**Affiliations:** Research Group “Alimentación y Nutrición en la Promoción de la Salud” (Food and Nutrition in Health Promotion [CEU-NutriFOOD]), Departamento de Ciencias Farmacéuticas y de la Salud, Facultad de Farmacia, Universidad San Pablo-CEU, CEU Universities, Urbanización Montepríncipe, 28660 Boadilla del Monte, Spain; Department of Preventive Medicine and Public Health, Universidad Autónoma de Madrid, 28029 Madrid, Spain; CIBER of Epidemiology and Public Health, Instituto de Salud Carlos III, 28029 Madrid, Spain; Research Group “Alimentación y Nutrición en la Promoción de la Salud” (Food and Nutrition in Health Promotion [CEU-NutriFOOD]), Departamento de Ciencias Farmacéuticas y de la Salud, Facultad de Farmacia, Universidad San Pablo-CEU, CEU Universities, Urbanización Montepríncipe, 28660 Boadilla del Monte, Spain; Research Group “Alimentación y Nutrición en la Promoción de la Salud” (Food and Nutrition in Health Promotion [CEU-NutriFOOD]), Departamento de Ciencias Farmacéuticas y de la Salud, Facultad de Farmacia, Universidad San Pablo-CEU, CEU Universities, Urbanización Montepríncipe, 28660 Boadilla del Monte, Spain

**Keywords:** appetite, CEBQ, energy intake, eating behavior, FFQ, rs17782313, SACINA, PFS, TFEQ-51, VAS

## Abstract

**Context:**

The melanocortin-4 receptor gene (MC4R) is associated with a higher risk of obesity by the presence of the C allele in rs17782313, but the mechanisms are not clear.

**Objective:**

The present systematic review and meta-analysis aimed to explore the association between the different genotypes of MC4R rs17782313 and energy intake and appetite.

**Data Sources:**

A literature search was conducted up to June 2023 in PubMed, Scopus, Web of Science, and Cochrane Collaboration databases, following PRISMA guidelines.

**Data Extraction:**

Inclusion criteria were studies in humans measuring energy intake, appetite, or satiety in all ages and physiological conditions. Studies dealing solely with body mass index were excluded. Twenty-one articles representing 48 560 participants were included in the meta-analysis.

**Data Analysis:**

According to the NHLBI (National Heart, Lung, and Blood Institute) quality-assessment criteria, all case-control studies and 6 out of 17 cohort and cross-sectional studies were classified as “good,” while the rest scored as “fair.” Odds ratios (ORs) and 95% confidence intervals (CIs) were calculated in a (CT+CC) vs TT dominant model, and both random-effects and fixed-effects models were used. A statistically significant association between the presence of the C allele and increased appetite was found (OR = 1.25; 95% CI: 1.01–1.49; P = .038) using the fixed-effects model, but the random-effects model proved nonsignificant. However, no association with energy intake was found. None of the variables considered (sample size, year of publication, sex, age group, type of population, origin, and quality) were identified as effect modifiers, and no publication biases were found after subgroup and meta-regression analyses.

**Conclusion:**

To our knowledge, this is the first systematic review and meta-analysis that has analyzed the association between rs17782313 of MC4R and energy intake and appetite. Identifying people genetically predisposed to increased appetite may be of great interest, not only to prevent obesity in younger populations but also to avoid malnutrition in elderly persons. This paper is part of the Nutrition Reviews Special Collection on Precision Nutrition.

**Systematic Review Registration:**

PROSPERO registration no. CRD42023417916.

## INTRODUCTION

The melanocortin-4 receptor gene (*MC4R*) has so far been associated with an increase in body mass index (BMI) and risk of obesity. The C allele of the single nucleotide polymorphism (SNP) rs17782313, a variant of this gene, is considered a risk factor for developing obesity.^1–3^ However, the mechanisms underlying this association have not been elucidated. One of the possible reasons could be that those with the obesity-associated genotype have a greater appetite and higher dietary consumption, leading to accumulation of energy in excess, which is reported as an increase in body weight.^4^ But, to date, this question has not been addressed. However, knowing whether this risk of obesity is due to an increase in appetite, and therefore to an increase in energy consumption, because of the genetic inheritance may have multiple benefits. It may help to identify people at a greater risk of developing obesity from an early age, and thus promote interventions aimed at obesity prevention through education on hunger-satiety mechanisms and dietary habits. On the other hand, in older people, having this genetic variant that is associated with increased appetite could be understood as protection against age-induced malnutrition.

Appetite is defined as “a desire for food”^5^ and satiety is defined as “the quality or state of being fed or gratified to or beyond capacity.”^6^ The balance between appetite and satiety is regulated by the hypothalamus. There is an anabolic pathway that is responsible for weight maintenance or weight gain inducing hunger and appetite signals. Likewise, there is a catabolic system in charge of weight maintenance or weight loss through activation of the gastrointestinal system and satiety signals. The hormones leptin and ghrelin play an important role in this regulation. These hormones transmit information on nutritional status to the central nervous system and have opposite functions: leptin inhibits food cravings, whereas ghrelin increases appetite. However, these hunger and satiety mechanisms can become unbalanced because of diets rich in ultra-processed foods, eating disorders, or obesity, among others.^7^^,^^8^

There are many factors that play an important role in appetite and energy intake, such as the environment, physical activity, or dietary habits. In addition, scientific evidence increasingly shows more importance of genetics, and specifically of nutrigenetics—that is, the way in which individuals react to dietary components, according to their genetic makeup. These new studies offer the possibility of personalizing nutrition according to the individual’s genetic profile.^9^ Taken together, it can be deduced that nutritional requirements are not the same for every person. Some of this individual variability is due to differences in body size, age, sex, physical activity, or health status, among others, but there is a significant residual variation that is attributed to genetic differences. In this sense, the *MC4R* gene is an important regulator of energy homeostasis, food intake, and body composition.^10^ This gene is located on chromosome 18q21 and encodes a 332 amino acid protein. It belongs to a family of membrane receptors that activate the response to melanocortin.^11^ Melanocortin plays an important role in the regulation of satiety and, consequently, in energy consumption.

Some studies have shown that the rs17782313 SNP of *MC4R* is related to differences in macronutrient intakes. Higher fat intake was reported in men with the *CT*/*CC* genotypes.^12^ Also, it has been found that people with the C allele who follow a low-protein and a high-energy and high-fat diet present higher BMI values, and are more likely to develop overweight or obesity.^13^ Previous studies have demonstrated that the *CC* genotype is associated with higher energy and lower carbohydrate and protein intakes^14^; however, others have found no differences in macronutrient intakes within genotypes.^15^

Most of the published studies focus on the association between the different genotypes of rs17782313 and BMI, regardless of energy intake. Some of them reported on different eating behaviors depending on the genotype through different questionnaires, such as the Child Eating Behavior Questionnaire (CEBQ) or the Three Factors Eating Questionnaire (TFEQ) to determine the association with weight gain and obesity.

However, to our knowledge, no study has separately reported a pooled estimate of the effect of the rs17782313 SNP on appetite and satiety. Those studies that have measured appetite have mostly used visual analogue scales (VASs) in the context of seeking an association with overeating behaviors, depression, obesity, and dietary patterns. But none have focused on whether people with higher BMI or obesity present higher appetite and energy intake according to their genotype. Therefore, the aim of this systematic review and meta-analysis was to investigate how the presence of 1 genotype or another of the rs17782313 SNP in the *MC4R* gene affects energy intake and appetite.

## METHODS

### Protocol and registration

The review was carried out following the Preferred Reporting Items for Systematics Reviews and Meta-Analyses (PRISMA) guidelines.^16^ It was registered in the International Database of Prospectively Registered Systematic Reviews (PROSPERO) as CRD42023417916.

### Search strategy

**Figure 1.**
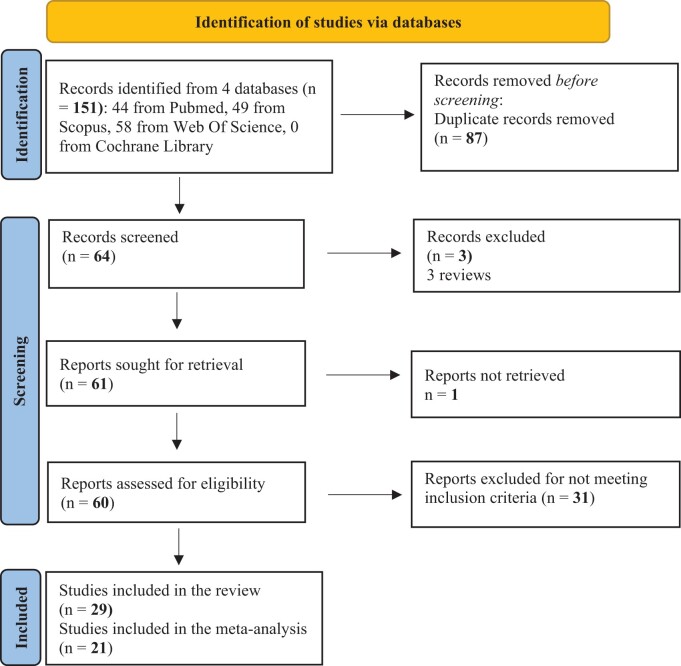
PRISMA 2020 flow diagram for the Selection of Studies

The literature search was performed from February 2023 to June 2023 using PubMed, Scopus, Web of Science, and Cochrane Collaboration databases. The review was conducted following the PRISMA 2020 flow diagram (Figure 1). Initially, 2 researchers (C.A.-M. and R.d.l.I.) working independently identified a total of 151 records from the 4 databases. Following the removal of duplicate studies, the titles and abstracts of the selected articles were thoroughly examined. Duplicates were identified using RefWorks bibliography manager and manually rechecked. Afterwards, the same 2 researchers examined the included and excluded studies to confirm the reason behind each decision in accordance with the inclusion criteria. In case of disagreement, a third researcher (E.A.-A.) was consulted for resolution. Eligible articles were those that examined the influence of the rs17782313 genotypes on appetite, satiety, and energy intake. The search strategy for the systematic review was carried out using the PICOS (Population, Intervention, Comparison, Outcome, and Study design) criteria, as detailed in Table 1. Search strategies in the databases can be found in Tables S1–S4. Moreover, when necessary, the authors of the articles included were contacted to obtain unpublished data needed to conduct the meta-analysis.

**Table 1.** PICOS Criteria for Inclusion of Studies

Parameter	Criteria
Participants	Humans of all ages and physiological situations
Intervention/exposure	*TC* and *CC* genotypes of the *MC4R* single nucleotide polymorphism rs17782313
Control/comparison	*TT* genotype of the *MC4R* single nucleotide polymorphism rs17782313
Outcome	Energy intake, appetite, and satiety
Study design	Observational studies including prospective cohort, case-control, and cross-sectional studies

*Abbreviation: MC4R*, melanocortin-4 receptor gene.

### Eligibility criteria

To be included in the review, articles needed to meet the following inclusion criteria: (1) studies in humans of all ages and physiological situations; (2) studies that examined the SNP rs17782313; (3) studies measuring energy intake, appetite, or satiety; and (4) studies written in Spanish or English.

The exclusion criteria were as follows: (1) studies in which the only outcome was obesity or any change in the BMI; (2) studies in which the only outcome was the effect on a chronic degenerative disease; (3) studies that do not provide data on energy intake, appetite, or satiety; and (4) studies written in a language other than English or Spanish.

### Quality assessment

The risk of bias in the studies was assessed using the tools of the National Heart, Lung, and Blood Institute (NHLBI): Quality Assessment Tool for Observational Cohort and Cross-sectional Studies and Quality Assessment of Case-Control Studies.^17^ One author (C.A.-M.) conducted the first approach and results were verified independently by 2 other researchers (R.d.l.I. and E.A.-A.).

The scale for observational cohort and cross-sectional studies examines bias through 14 questions on the following: (1) research question, (2 and 3) study population, (4) groups recruited from the same population and with uniform eligibility criteria, (5) sample size justification, (6) exposure assessed prior to outcome measurement, (7) sufficient time frame to see an effect, (8) different levels of the exposure of interest, (9) exposure measures and assessment, (10) repeated exposure assessment, (11) outcome measures, (12) blinding of outcome assessors, (13) follow-up rate, and (14) statistical analyses. In the same way, the tool for case-control studies has only 12 questions but, in terms of content, is very similar to the previous one: (1) research question, (2) study population, (3) sample size justification, (4) groups recruited from the same population, (5) inclusion and exclusion criteria prespecified and applied uniformly, (6) case and control definitions, (7) random selection of study participants, (8) concurrent controls, (9) exposure assessed prior to outcome measurement, (10) exposure measures and assessment, (11) blinding of exposure assessors, and (12) statistical analysis. In both scales, each item of methodological quality is classified as “yes,” “no,” “cannot be determined,” “not applicable,” or “not reported,” and based on the number of “yes” as a total score, studies were classified according to quality rating as poor (<50%), fair (50%–75%), and good (>75%).^18^

### Data-collection process

Data were systematically extracted and included the following: study author(s), year and country of publication, study design, age distribution, sample size, type of population, measured variables (ie, genotype, energy intake or appetite and/or satiety), and instruments used to measure the variables. The first author (C.A.-M.) conducted the extraction, and the accuracy of the extracted data was verified by all authors (R.d.l.I., E.A.-A., F.F.C.). No automation tool was used during the process.

To carry out the statistical analysis, the following data were obtained from the original articles: sample size, mean and SD of energy intake or appetite measures for each genotype (*TT*, *CT*, and *CC*). Then, odds ratios (ORs) and 95% confidence intervals (CIs) were calculated for 3 different models, using the *TT* genotype as the reference category: *CT* vs *TT*, *CC* vs *TT*, and (*CT*+*CC*) vs *TT*. Finally, it was decided to conduct a dominant model, in which a single copy of the C allele is sufficient to modify risk. Therefore, the combination of the 2 genotypes with the minority allele (*CT* and *CC*) with respect to *TT* homozygotes was compared.

Missing data were handled by contacting study investigators for unreported data or additional details. Studies with incomplete information were not included in the meta-analysis, but they are discussed in the systematic review. All data were compiled in a Microsoft Excel spreadsheet (Microsoft Corporation, Redmond, WA, USA).

### Data synthesis

The data from the 29 selected studies were classified according to the variable measured—that is, energy intake or appetite. In the case of appetite, 3 researchers (C.A.-M., R.d.l.I., and E.A.-A.) evaluated the different questionnaires on eating behavior used in the studies. Finally, the variables selected were “susceptibility to hunger” from the TFEQ (TFEQ-51) and “food responsiveness” from the CEBQ. In addition, studies that measured appetite using VASs and/or Power Food Scales (PFSs) were also included.

Of a total of 29 studies, the authors of 21 of them were contacted to ask for necessary data, such as sample size and mean or SD of the main variable. Consequently, ORs and 95% CIs for each model were calculated using the Campbell Collaboration Calculator.^19^

### Statistical analysis

A total of 21 studies were included in the meta-analysis. Data were analyzed with STATA 15.0 software (StataCorp, College Station, TX, USA) using the *metan*, *metareg*, and *metabias* commands. Odds ratios and 95% CIs were considered for the meta-analysis. A main meta-analysis with the quantitative variable “energy intake” (kcal/day) and a second one with the results of the studies that had measured appetite using VASs, PFSs, and eating behavior questionnaires (TFEQ-51 and CEBQ) was carried out. To avoid potential issues related to heterogeneity, a random-effects model was proposed as an alternative to the fixed-effects model. The inverse variance–weighted method^20^ was used to obtain an overall effect size and 95% CI; in this approach, the weight given to each study is the inverse of the variance of the effect estimate. Thus, larger studies are given higher weight than smaller studies, which have larger standard errors. This type of weight consideration can minimize the imprecision of the pooled effect estimate.

The heterogeneity was assessed by means of the Cochran’s Q test and quantified by the *I^2^* statistic, which measures the proportion of the total variation due to heterogeneity. When *I^2^* was greater than 50%, the existence of heterogeneity was considered.^21^ A random-effects meta-regression was used to explore sources of heterogeneity and to identify study characteristics that may influence the association between genotype and energy intake or appetite. In a first step, the following variables were considered as potential confounders: sample size, year of publication (before and after 2018), sex (both sexes and only females), age group (children, teenagers, and adults), type of population (overweight and obese or general), origin (European, American, and Asian), and quality rating (poor, fair, and good). The univariate association between each of these variables considered and energy intake and appetite was assessed. When the *P-*value was less than .05, confounder variables were respectively included in the meta-regression analysis conducted for energy intake and appetite.

Potential publication bias was assessed separately for the meta-analysis of energy intake and appetite, by the application of Egger's linear regression test.^22^ Egger's test examines whether the association between estimated intervention effects and a measure of study size (such as the standard error of the intervention effect) is greater than might be expected to occur by chance. A funnel plot was constructed by plotting the effect measure against the inverse of its standard error. An asymmetric plot indicates a likely publication bias and *P *<* *.05 for the estimated intercept in the Egger's linear regression model is considered representative of statistically significant publication bias.

## RESULTS

### Study selection and study characteristics

The systematic review and meta-analysis flow is presented in Figure 1. One hundred and fifty-one studies were included: 44 from PubMed, 49 from Scopus, and 58 from Web of Science. After deleting duplicate records, a total of 64 studies remained. Four of them were excluded, because they were reviews (3 studies) or because the full text was unretrievable (1 study). Of the remaining 60 studies, 31 had to be excluded because they did not meet the inclusion criteria. Finally, a total of 29 studies were included in the review. These studies were published between 2009 and 2023. Most of them were cross-sectional, except for a small number of cohort studies (4) and case-control studies (4). Of 29 studies, a total of 21 were included in the meta-analysis: 16 for the meta-analysis on energy intake and 7 for the meta-analysis on appetite. In the meta-analysis conducted for appetite, 2 different articles were considered as the same study since they were both conducted in the same population group and the author provided us with the dataset for both. Eight studies were excluded from the meta-analysis due to insufficient data and lack of response from the authors after attempting to reach them during a 4-month period. Table 2 summarizes the baseline characteristics of the studies included in the systematic review.^12–15^^,^^23–47^

**Table 2.** Characteristics of Studies Included in the Systematic Review and/or Meta-analysis

Code	Study (year)	Country	Study design	Age (years) distribution and sex	Sample size, total (genotyped)	Type of population	Variables	Instrument
1	Stutzmann et al (2009)^23^	France, Switzerland, and Finland	Cross-sectional	18–89 Both sexes	*n* = 17 527 (NA)	Adolescents and adults with and without obesity (BMI ≥30 kg/m^2^)	Eating behavior	51-item TFEQ
2	Hasselbalch et al (2010)^24^	Denmark	Cross-sectional	18–67 Both sexes	*n* = 1512 (1115) *TT* (*n* = 668), *CT* (*n* = 402), *CC* (*n* = 45)	Healthy adult twin pairs	Energy intake	247-item FFQ
3	Taylor et al (2011)^25^	India	Cross-sectional	20–69 Both sexes	*n* = 6780 (6466) *TT* (*n* = 2724), CT (*n* = 2907), CC (*n* = 835)	Healthy adults	Energy intake	184-item FFQ
4	Corella et al (2012)^26^	Spain	Cross-sectional	Males: 55–80 Females: 60–80 Both sexes	*n* = 7447 (7219) *TT* (*n* = 4336), *CT* (*n* = 2553), *CC* (*n* = 330)	Adults with type 2 diabetes and/or ≥3 cardiovascular risk factors (hypertension, dyslipidemia, BMI ≥25 kg/m^2^, current smoking, or a family history of premature cardiovascular disease)	Energy intake	137-item semi-quantitative FFQ
5	Horstmann et al (2013)^27^	Germany	Cross-sectional	24–29 Both sexes	*n* = 221 (221) *TT* (*n* = 119), *CT* (*n* = 87), *CC* (*n* = 15)	Healthy adults	Eating behavior	51-item TEFQ
6	Valladares et al (2010)^28^	Chile	Cross-sectional	5–15 Both sexes	*n* = 221 (148) *TT* (*n* = 105), *CT* (*n* = 39), *CC* (*n* = 4)	Children and adolescents with overweight and obesity	Eating behavior	CEBQ
7	Acosta et al (2014)^29^	USA	Cross-sectional	18–65 Both sexes	*n* = 178 (178) *TT* (n = 94), *CT* (*n* = 72), *CC* (*n* = 12)	Healthy White adults with overweight and obesity (BMI > 25 kg/m^2^)	Satiety	Nutrient drink test and ad libitum meal
8	Ho-Urriola et al (2014)^30^	Chile	Case-control	6–12 Both sexes	*n* = 377 (377) Cases (*n* = 238): TT (*n* = 173), *CT* (*n* = 60), *CC* (*n* = 5) Controls (*n* = 139): *TT* (*n* = 110), *CT* (*n* = 26), *CC* (*n* = 3)	Cases: children with overweight and obesity Controls: children with normal weight	Eating behavior	CEBQ
9	Katsuura-Kamano et al (2014)^31^	Japan	Cross-sectional	35–69 Both sexes	*n* = 2035 (2035) *TT* (*n* = 1268), *CT* (*n* = 669), *CC* (*n* = 98)	General population	Energy intake	47-item FFQ
10	Dušátková et al (2015)^32^	Czech Republic	Cross-sectional	10–18 Both sexes	*n* = 1953 (NA)	Children and adolescents with normal weight (BMI < 90th percentile) and with overweight/obesity (BMI ≥ 90th percentile)	Energy intake	3-day food record
11	Khalilitehrani et al (2015)^14^	Iran	Cross-sectional	>22 Both sexes	*n* = 400 (374) *TT* (*n* = 156), *CT* (*n* = 111), *CC* (*n* = 107)	Healthy adults	Energy intake	3-day food record
12	Yilmaz et al (2015)^33^	Canada	Cross-sectional	24–50 Both sexes	*n* = 328 (280) *TT* (*n* = 168), *CT* (*n* = 92), *CC* (*n* = 20)	Healthy adults	Appetite	PFS
13	Lauria et al (2016)^34^	Belgium, Cyprus, Estonia, Germany, Hungary, Italy, Spain, and Sweden	Prospective cohort	2–9 Both sexes	*n* = 16 228 (1941) *TT* (*n* = 1138), *CT* (*n* = 697), *CC* (*n* = 106)	Schoolchildren	Energy intake	SACINA
14	Park et al (2016)^15^	Korea	Cross-sectional	40–69 Both sexes	*n* = 8842 (8830) *TT* (*n* = 5033), *CT* (*n* = 3246), *CC* (*n* = 551)	Adults from rural and urban communities	Energy intake	103-item FFQ
15	Obregon et al (2017)^35^	Chile	Cross-sectional	8–14 Both sexes	*n* = 258 (256) *TT* (*n* = 198), *CT* (*n* = 56), *CC* (*n* = 2)	Children with obesity, overweight, and normal weight	Eating behavior	CEBQ
16	Martins et al (2018)^36^	Brazil	Prospective cohort	20–40 Females	*n* = 149 (143) *TT* (n = 93), *CT* (*n* = 46), *CC* (*n* = 4)	Obese midterm pregnant females	Energy intake	Semi-quantitative FFQ
17	Meng et al (2018)^37^	USA	Prospective cohort	≥18 Females	*n* = 85 (79) *TT* (*n* = 43), *CT* (*n* = 32), *CC* (*n* = 4)	Pregnant females and mothers with children up to 6 months with BMI between 18.5 kg/m^2^ and 40 kg/m^2^	Energy intake	24-hour dietary recall
18	Adamska-Patruno et al (2019)^38^	Poland	Cross-sectional	18–65 Both sexes	*n* = 927 (927) *TT* (*n* = 584), *CT* (*n *= 316), *CC* (*n* = 27)	Adults with overweight/obesity (BMI ≥25 kg/m^2^) and with normal weight (BMI <25 kg/m^2^)	Energy intake	3-day food record
19	Mohammadi et al (2020)^39^	Iran	Cross-sectional	20–50 Both sexes	*n* = 288 (288) *TT* (*n* = 114), *CT* (*n* = 96), *CC* (*n* = 78)	Apparently healthy adults with obesity (BMI between 30 and 40 kg/m^2^)	Energy intake (a)	132-item semi-quantitative FFQ
							Appetite (b)	VAS
20	Mousavizadeh et al (2020)^40^	Iran	Prospective cohort	≥18 Both sexes	*n* = 3850 (NA)	General population	Energy intake	168-item semi-quantitative FFQ
21	Khodarahmi et al (2020)^41^	Iran	Cross-sectional	20–50 Both sexes	*n* = 188 (141) *TT* (*n* = 63), *CT* (*n* = 51), *CC* (*n* = 27)	Apparently healthy adults with obesity (BMI ≥30 kg/m^2^)	Energy intake (a)	147-item semi-quantitative FFQ
							Appetite (b)	VAS
22	Magno et al (2021)^42^	Brazil	Case-control	20–48 Females	*n* = 70 (70) Cases (*n* = 26): *CT* (*n* = 22), *CC* (*n* = 4) Controls: *TT* (*n* = 44)	Females with °BMI between 40 and 60 kg/m^2^ and who have had obesity for at least 5 years Cases: *CT/CC* genotypes Controls: *TT* genotype	Energy intake (a)	3-day food record
							Appetite (b)	VAS
							Ghrelin and leptin (c)	Blood analysis
23	Narjabadifam et al (2021)^43^	Iran	Case-control	17–59 Females	*n* = 563 (563) Cases (*n* = 396): *TT* (*n* = 144), *CT* (*n* = 192), *CC* (*n* = 60) Controls (*n* = 167): *TT* (*n* = 72), *CT* (*n* = 80), *CC* (*n* = 15)	Cases: females with obesity and overweight (BMI ≥ 25 kg/m^2^) Controls: females with BMI < 25 kg/m^2^	Hedonic hunger	PFS
24	Raskiliene et al (2021)^12^	Lithuania	Prospective cohort	12–13 Both sexes	*n* = 1082 (503) *TT* (*n* = 343), *CT* (*n* = 152), *CC* (*n* = 8)	Schoolchildren	Energy intake	24-hour dietary recall
25	Alizadeh et al (2022)^44^	Iran	Cross-sectional	18–56 Females	*n* = 282 (NA) *TT* (*n* = 153)	Healthy females with overweight/obesity (BMI between 25.2 and 49.60 kg/m^2^)	Energy intake	147-item FFQ
26	Rahati et al (2022)^13^	Iran	Cross-sectional	20–50 Both sexes	*n* = 403 (403) *TT* (*n* = 100), *CT* (*n* = 250), *CC* (*n* = 53)	Healthy adults with overweight or obesity (BMI between 25 and 40 kg/m^2^)	Energy intake (a)	3-day food record
							Appetite (b)	VAS
27	Nacis et al (2022)^45^	Philippines	Cross-sectional	13–18 Both sexes	*n* = 280 (280) *TT* (*n* = 230), *CT* (*n* = 49), *CC* (*n* = 1)	Healthy adolescents	Energy intake	5-day food record
28	Zarei et al (2022)^46^	Iran	Cross-sectional	18–48 Females	*n* = 291 (275) *TT* (*n* = 83), CT (*n* = 69), *CC* (*n* = 123)	Females with overweight/obesity (BMI ≥25 kg/m^2^)	Energy intake	147-item FFQ
29	Rasaei et al (2023)^47^	Iran	Cross-sectional	18–68 Females	*n* = 378 (NA)	Females with overweight or obesity (BMI of 25–40 kg/m^2^)	Energy intake	147-item FFQ

*Abbreviations:* BMI, body mass index; CEBQ, Child Eating Behavior Questionnaire; FFQ, food-frequency questionnaire; NA, not available; PFS, Power Food Scale; SACINA, Self-Administered Children and Infant Nutrition Assessment; TFEQ, Three Factors Eating Questionnaire; VAS, visual analogue scale.

### Risk of bias within studies

According to the Quality Assessment Tool for Observational Cohort and Cross-sectional Studies of the NHLBI,^17^ 64.7% of the analyzed studies were classified as “fair quality” studies and the remaining 35.3% as “good quality” studies (Table 3).^12–15^^,^^24–26^^,^^31^^,^^33–38^^,^^44–46^ Case-control studies (Table 4)^28^^,^^30^^,^^42^^,^^43^ were evaluated through the Quality Assessment of Case-Control Studies of the NHLBI,^17^ and all of them were classified as “good quality” studies (100%).

**Table 3.** Quality-Assessment Tool for Observational Cohort and Cross-sectional Studies

		Questions evaluated															
Code	Study (year)	1	2	3	4	5	6	7	8	9	10	11	12	13	14	Total score^a^	Quality rating
2	Hasselbalch et al (2010)^24^	Yes	NR	Yes	Yes	No	No	No	NA	Yes	NA	Yes	NA	NA	Yes	6/9 (66.67%)	Fair
3	Taylor et al (2011)^25^	Yes	NR	Yes	No	No	No	No	NA	Yes	NA	Yes	NA	NA	Yes	5/9 (55.56%)	Fair
4	Corella et al (2012)^26^	Yes	Yes	Yes	Yes	Yes	No	No	NA	Yes	NA	Yes	NA	NA	Yes	8/10 (80%)	Good
9	Katsuura-Kamano et al (2014)^31^	Yes	NR	Yes	Yes	No	No	No	NA	Yes	NA	Yes	NA	NA	Yes	6/9 (66.67%)	Fair
11	Khalilitehrani et al (2015)^14^	Yes	Yes	Yes	NR	No	No	No	NA	Yes	NA	Yes	NA	NA	Yes	6/9 (66.67%)	Fair
12	Yilmaz et al (2015)^33^	Yes	Yes	Yes	Yes	No	No	No	NA	Yes	NA	Yes	NA	NA	Yes	7/10 (70%)	Fair
13	Lauria et al (2016)^34^	Yes	Yes	Yes	NR	Yes	Yes	Yes	NA	Yes	NA	Yes	NA	Yes	Yes	10/10 (100%)	Good
14	Park et al (2016)^15^	Yes	Yes	Yes	Yes	No	No	No	NA	Yes	NA	Yes	NA	NA	Yes	7/10 (70%)	Fair
15	Obregon et al (2017)^35^	Yes	Yes	Yes	Yes	Yes	No	No	NA	Yes	NA	Yes	NA	NA	No	7/10 (70%)	Fair
16	Martins et al (2018)^36^	Yes	Yes	Yes	Yes	Yes	Yes	Yes	NA	Yes	NA	Yes	NA	Yes	Yes	11/11 (100%)	Good
17	Meng et al (2018)^37^	Yes	Yes	Yes	Yes	No	Yes	Yes	NA	Yes	NA	Yes	NA	Yes	Yes	10/11 (90.91%)	Good
18	Adamska-Patruno et al (2019)^38^	Yes	NR	Yes	Yes	No	No	No	NA	Yes	NA	Yes	NA	NA	Yes	6/9 (66.67%)	Fair
24	Raskiliene et al (2021)^12^	Yes	Yes	Yes	Yes	No	Yes	Yes	NA	Yes	NA	Yes	NA	No	Yes	9/11 (81.82%)	Good
25	Alizadeh et al (2022)^44^	Yes	Yes	Yes	Yes	Yes	No	No	NA	Yes	NA	Yes	NA	NA	Yes	8/10 (80%)	Good
26	Rahati et al (2022)^13^	Yes	Yes	Yes	Yes	No	No	No	NA	Yes	NA	Yes	NA	NA	Yes	7/10 (70%)	Fair
27	Nacis et al (2022)^45^	Yes	Yes	Yes	Yes	No	No	No	NA	Yes	NA	Yes	NA	NA	No	6/10 (60%)	Fair
28	Zarei et al (2022)^46^	Yes	Yes	Yes	Yes	No	No	No	NA	Yes	NA	Yes	NA	NA	Yes	7/10 (70%)	Fair

*Abbreviations:* NA, not applicable; NR, not reported.

a Total score: number of “yes.” Quality rating: poor <50%; fair: 50%–75%; good >75%.

**Table 4.** Quality Assessment of Case-Control Studies

		Questions evaluated													
Code	Study (year)	1	2	3	4	5	6	7	8	9	10	11	12	Total score^a^	Quality rating
6 + 8^b^	Valladares et al (2010)^28^ + Ho-Urriola et al (2014)^30^	Yes	Yes	Yes	Yes	NR	Yes	NA	No	Yes	Yes	NR	Yes	8/9 (88.89%)	Good
22	Magno et al (2021)^42^	Yes	Yes	No	Yes	Yes	Yes	NA	No	Yes	Yes	NR	Yes	8/10 (80%)	Good
23	Narjabadifam et al (2021)^43^	Yes	Yes	No	Yes	Yes	Yes	NA	No	Yes	Yes	NR	Yes	8/10 (80%)	Good

*Abbreviations:* NA, not applicable; NR, not reported.

a Total score: number of “yes.” Quality rating: poor <50%; fair: 50%–75%; good >75%.

b Studies 6 and 8 were considered as 1 study since they were both conducted in the same population group.

A total of 43.75% of studies included in the energy intake synthesis were classified as “good quality.” Most of the studies that were classified as “fair quality” failed in the questions related to sample size justification, time of exposure assessment, and time frame to see an effect. This is because they were cross-sectional studies. In case of appetite synthesis, 50% were classified as “good quality” studies and 50% as “fair quality,” due to the same circumstances as the studies included in the energy intake synthesis.

### Meta-analysis results

**Figure 2.**
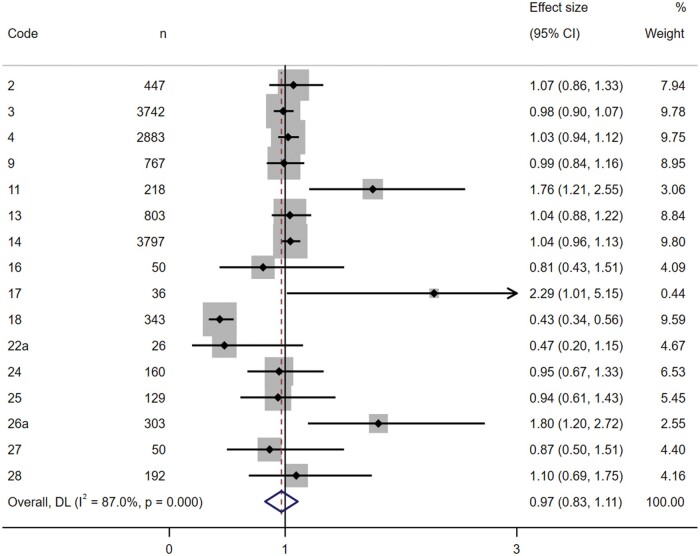
Forest Plot of Random-Effects Meta-analysis for the Relationship Between the C-Allele Carriers of *MC4R* rs17782313 and Energy Intake. *Abbreviations:* DL, DerSimonian-Laird method to estimate between study variance; *MC4R*, melanocortin-4 receptor gene

To carry out the analyses, a dominant model focused on the comparison between the *CT*+*CC* and the *TT* genotypes was conducted. All of the studies that assessed energy intake were collectively analyzed in the main meta-analysis. Given the high heterogeneity observed (*I^2^* = 87%), a random-effects meta-analysis was conducted (Figure 2). The pooled effect revealed a decrease in energy intake among individuals carrying the C allele (OR = 0.97; 95% CI: 0.83–1.11). However, due to the substantial heterogeneity observed, this effect did not reach statistical significance (*P = *.660). Nonetheless, there was a consistent trend indicating an association between the C allele and reduced energy intake. In fact, the less conservative fixed-effects model showed a significant pooled effect (OR = 0.93; 95% CI: 0.89–0.98; *P *=* *.001).

**Figure 3.**
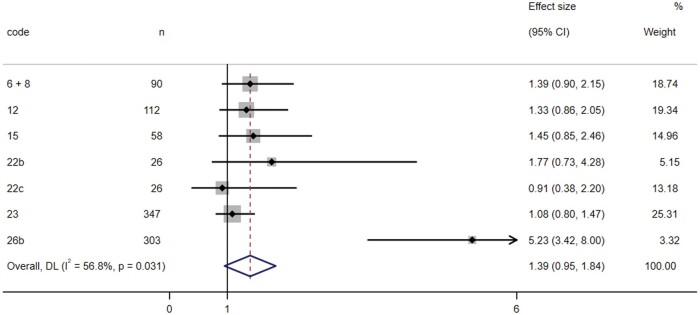
Forest Plot of Random-Effects Meta-analysis for the Relationship Between the C-Allele Carriers of *MC4R* rs17782313 and Appetite. *Abbreviations:* DL, DerSimonian-Laird method to estimate between study variance; *MC4R*, melanocortin-4 receptor gene

Second, the studies were collectively analyzed in a meta-analysis for appetite (Figure 3). In this case, heterogeneity was lower (*I^2^* = 56.8%), although also suggested the use of a random-effects meta-analysis. The combined result indicated a higher appetite in individuals with the C allele (OR = 1.39; 95% CI: 0.95–1.84), but this result was not statistically significant (*P *=* *.089). When using a fixed-effects meta-analysis, a statistically significant association was found between the presence of the C allele and an increased appetite (OR = 1.25; 95% CI: 1.01–1.49; *P *=* *.038). Individuals with the *CC* or *CT* genotype exhibited a greater appetite compared with those with the *TT* genotype.

Due to the results found in the main meta-analysis for energy intake, different variables were tested as effect modifiers: sample size, year of publication, sex, age group, type of population, origin, and quality. A univariate analysis was carried out for each of these variables and, when statistically significant (*P *<* *.05), the potential confounders were included in a meta-regression model (Table 5). No statistically significant differences were found for the variables considered; therefore, none of the variables seemed to modify the effect. As a result of the limited number of studies, it was not feasible to conduct the meta-regression analyses for appetite.

**Table 5.** Meta-regression Analysis for Confounding Variables in the Relationship Between the C-Allele Carriers of the *MC4R* rs17782313 and Energy Intake

	Coefficient	*P*	95% CI	
Sample size	−0.068	.336	−0.220	0.084
Year of publication (>2018)	0.337	.153	−0.152	0.827
Sex (females)	0.366	.343	−0.461	1.192
Type of population (overweight and/or obesity)	−0.275	.439	−1.045	0.494
Origin (European)	0.156	.200	−0.099	0.412
Quality (fair)	0.195	.371	−0.274	0.665

**Figure 4.**
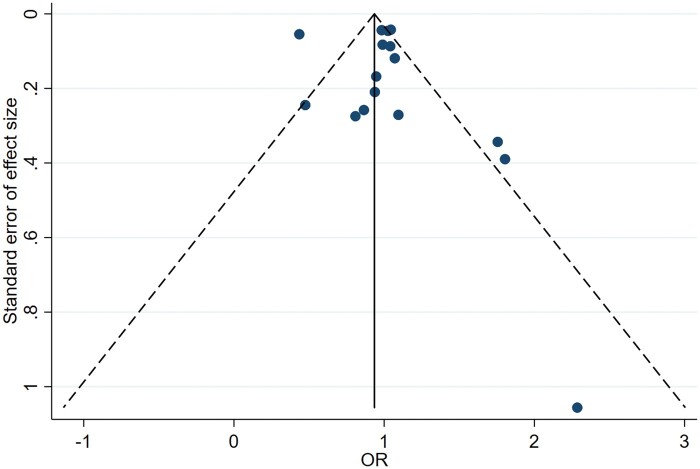
Funnel Plot for Energy Intake, Representing the Effect Size Against Its Standard Error. *Abbreviation:* OR, odds ratio

Egger's linear regression suggested that publication bias and small-study effects were not found: the estimated intercept for the fitted regression model for energy intake was 0.549 with a standard error of 1.125, giving a nonsignificant *P*-value (*P = *.633). The result is also illustrated by means of a funnel plot in Figure 4, where the funnel plot appears symmetric. Accordingly, it can be concluded that there is no publication bias.

**Figure 5.**
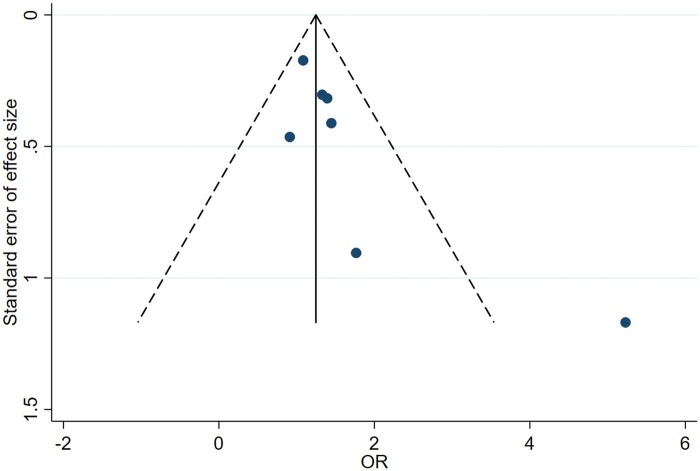
Funnel Plot for Appetite, Representing the Effect Size Against Its Standard Error. *Abbreviation:* OR, odds ratio

In the same way, the estimated intercept for the fitted regression model for appetite was 2.047, with a standard error of 0.882, giving a non-significant p-value (*P = *0.068). The funnel plot illustrated in Figure 5 again appears to seem symmetric, so there is no publication bias.

## DISCUSSION

To our knowledge, this is the first meta-analysis that has analyzed the association between the polymorphism rs17782313 of the *MC4R* gene and energy intake and appetite. The overall meta-analysis shows that appetite is associated with the C allele of rs17782313. Individuals carrying the *CC* or *CT* genotypes of this SNP have greater appetite compared with those who carry the *TT* genotype, which may explain why this variant has been associated with obesity in multiple studies.^2^ However, no association between the rs17782313 SNP and energy intake was found.

Appetite is regulated by the hypothalamus, where several neural centers participate in controlling food intake.^48^ Emotions are closely related to food and can affect appetite. Some studies have shown that the way you feel can induce changes in how you feed yourself. Negative emotions, such as fear or sadness, may increase impulsive eating and decrease food pleasantness, whereas boredom may be associated with increased appetite.^49^ Even though emotions play a role in eating behavior, studies show that there is an important influence of genetics in all of this regulation. For example, oxytocin, a 9-amino-acid peptide synthesized in the hypothalamus, has been associated with appetite. In particular, the G allele of the SNP rs53576 of the oxytocin receptor gene (*OXTR)* is associated with binge eating behaviors.^50^

Appetite and eating behavior are usually assessed through validated questionnaires. The VAS is one of the main rating scales, used for the first time in 1921 by Hayes and Patterson,^51^ to measure appetite. Studies also measured appetite through the PFS, a 21-item scale that was developed to assess the psychological influence of the availability of food.^52^

A study that investigated the association and interaction of the *MC4R* rs17782313 polymorphism with food intake and eating behavior found a significant association between the rs17782313 polymorphism and appetite: appetite in *TT* carriers of rs17782313 was significantly lower than that in *CT* and *CC* carriers. According to the VAS, C allele carriers had the lowest score.^13^ Together with the result on the association between rs17782313 polymorphism and appetite, other studies have also proven that genetics has an important influence on other effectors in the regulation of food intake, as well as on food-related emotions and how individuals are able to manage them. For example, different studies have found that CEBQ scores of “enjoyment of food” were higher and “satiety responsiveness” was lower in children with the *CC* genotype compared with *TT* homozygotes.^28^^,^^30^ Others have found that obese girls, carriers of the C allele, showed lower scores of “satiety responsiveness” and higher scores of “uncontrolled eating,” concluding that the C allele is associated with eating behavior traits that may predispose children with obesity to increased energy intake.^35^ Likewise, evidence shows that people with the *CC* genotype are more likely to show emotional eating behavior, which increases the risk of obesity.^13^ On the other hand, it has been shown that females with the C allele of the rs17782313 polymorphism have a higher prevalence of binge eating.^42^ Further studies have associated binge eating with current morbid obesity (BMI ≥40 kg/m^2^).^53^ Moreover, recent evidence suggests a positive interaction between the *CT* genotype of rs17782313 polymorphism and the food cholesterol–saturated fat index, indicating low-quality fat intakes, in people experiencing depression.^47^

Due to the substantial heterogeneity, the meta-analysis conducted for energy intake did not reach statistical significance. This result is in line with most of the studies included in the meta-analysis, because only 4 out of the 16 studies included found a significant association between the rs17782313 polymorphism of the *MC4R* gene and energy intake.^13^^,^^14^^,^^37^^,^^38^ A lack of association with energy intake is difficult to understand given that it is well evidenced that the C allele of the *MC4R* rs17782313 polymorphism is associated with obesity, as proven by the latest meta-analyses found in the literature.^2^^,^^3^ This may be due to different reasons. One of them could be the different populations studied. Most of the studies included in the meta-analysis were conducted in Asian populations and only 5 out of 16 studies included European White subjects. Therefore, it is possible that these results would be different if the majority of the populations were White. In addition, 25% of the studies were conducted in a population that had overweight and/or obesity from the beginning, which could bias the results on energy intake. In fact, 1 of the studies revealed a higher association between the *CC* genotype and energy intake in all participants, except for participants with a BMI < 25 kg/m^2^.^14^ Moreover, another study conducted only in participants with overweight or obesity found that *CC* genotype carriers had a higher intake of energy than *TT* carriers.^13^

In addition, it must be emphasized that studies measured energy intake through different self-reported questionnaires such as food-frequency questionnaires, Self-Administered Children and Infant Nutrition Assessment (SACINA), 24-dietary recalls, and food records, which may lead to variations in the results, and the difficulties in assessing precise dietary intake are well known.^53–56^ In addition, not having measured energy balance means that caution is warranted when making conclusions from results. If data are solely given for energy intake, the important role of energy expenditure and energy storage, which, together with energy intake, is essential for determining body weight, is overlooked. Therefore, the assessment of the association between rs17782313 and energy balance, which has not been addressed in previous studies, may provide different results. Moreover, it may not only be a question of energy imbalance but of macronutrient distribution. In this sense, higher intakes of fat and proteins, and lower intakes of carbohydrates, have been associated with *CC* genotypes in healthy individuals.^14^ Furthermore, people with the *CC* genotype and, in addition, with overweight and obesity tend to have lower intakes of carbohydrates.^13^

One of the latest meta-analyses on the association between polymorphisms and obesity showed that the *MC4R* rs17782313 polymorphism was significantly associated with obesity risk in children, but the exact pathway or mechanism underlying this association is not yet specified and requires further investigation.^57^ In view of these results, people with the *CC* genotype may tend to gain more weight because of a bigger appetite. High-quality studies as well as further study on the mechanisms involved are needed to assess whether an imbalance in macronutrient intake could also explain why *MC4R* rs17782313 is associated with obesity. In addition, rather than focusing on quantifying total energy intake, it may be advisable to assess energy balance to gain a clearer understanding of these associations.

Despite these novel findings, it is important to acknowledge some limitations. First, the 29 articles included in the review could not be included in the meta-analysis due to lack of data on sample size and mean or SD of the different genotypes for some of the studies. It was possible to contact the authors of 21 of the studies, who provided the data necessary to calculate the ORs and the 95% CIs for each genetic model, but unfortunately, the necessary information could not be retrieved from 8 of the studies, which consequently could not be included in the meta-analysis. Nevertheless, all of them were focused mainly on the analysis of the association of the rs17782313 polymorphism with the risk of obesity rather than with energy intake and appetite. Second, the heterogeneity in the studies may have biased the results. However, to avoid potential issues related to heterogeneity, a random-effects model was proposed as an alternative to the fixed-effects model. The random-effects modelling provides more conservative results than a fixed-effects model.^58^ Random-effects models consider the amount of variance caused by differences between studies, as well as differences among participants within studies. Likewise, funnel plots for energy intake and appetite were symmetrical and Egger’s linear regression test indicated no publication bias with any of the confounding variables included. Although population origin did not prove to be a confounding variable in the analysis, the bias produced by ancestry cannot be ruled out, since the categories used are not detailed enough to detect a possible effect.

This study has several strengths. First, this is the first systematic review and meta-analysis to provide a thorough summary of the association between the rs17782313 SNP of the *MC4R* gene, appetite, and energy intake. Previous meta-analyses have focused solely on the relationship between the SNP and obesity. The results reveal that the C allele of SNP rs17782313 is associated with appetite which may help identify populations at risk of future malnutrition. Second, the review was undertaken using a meticulous methodology. Strict inclusion criteria were adopted, and more than 48 000 participants were included in the meta-analysis. Combining data from many studies to form a large sample size allows small effects to be detected and more precise estimates to be obtained. Third, 2 meta-analyses, one with the variable “energy intake” and another with the variable “appetite,” were carried out in order to differentiate between the 2 characteristics and to see whether one could be the consequence of the other. Odds ratios with 95% CIs (under a dominant model) and a random-effects meta-regression were used to explore sources of heterogeneity. The potential publication bias was assessed separately for both meta-analyses, by the application of Egger's linear regression test, and no publication bias was detected.

## CONCLUSION

According to the results, appetite is associated with the C allele of the rs17782313 polymorphism in the *MC4R* gene, proving that genetic inheritance is of particular importance in regulating dietary intake, including psychological effectors such as appetite. Identifying people who may have a greater appetite due to their genotype could be of great help in the prevention of obesity. On the other hand, a genetically determined greater appetite could also be beneficial to older people, who usually decrease energy intake due to physiological changes and are prone to a higher risk of malnutrition. Nevertheless, further studies are needed to assess the relationship between eating behavior, appetite, and satiety in individuals with different genotypes of the rs17782313 SNP of *MC4R*.

## Supplementary Material

nuae075_Supplementary_Data

## Data Availability

Raw data and supplemental materials used in this study will be made publicly and freely available without restriction at the institutional repository at Universidad San Pablo-CEU (CEU Repositorio Institucional) at https://repositorioinstitucional.ceu.es/.

## Notes

This article is part of a Special Collection on Precision Nutrition.
