# Supplementary material for: Association of MC4R rs17782313 Genotype With Energy Intake and Appetite: A Systematic Review and Meta-analysis
Source: Nutr Rev. 2024 Jun 14;83(3):e931–46. doi: 10.1093/nutrit/nuae075 (PMC11819477; doi:10.1093/nutrit/nuae075)
Supplement: nuae075_Supplementary_Data [file nuae075_supplementary_data.zip › nuae075_Supplementary_Data/Supplementary tables.docx]

**Supplementary Tables**

**Supplementary Table 1**. Search strategy for Pubmed

| Search Set | Pubmed | Results |
| --- | --- | --- |
| #1 | (rs17782313 AND appetite) [All Fields] | 11 |
| #2 | (rs17782313 AND satiety) [All Fields] | 9 |
| #3 | (rs17782313 AND “energy intake”) [All Fields] | 16 |
| #4 | (rs17782313 AND “eating behaviour”) [All Fields] | 2 |
| #5 | (rs17782313 AND “eating behavior”) [All Fields] | 10 |
| #6 | (rs17782313 AND ghrelin) [All Fields] | 5 |
| #7 | (rs17782313 AND gluttony) [All Fields] | 4 |
| #8 | (rs17782313 AND hunger) [All Fields] | 6 |
| #9 | (rs17782313 AND leptin) [All Fields] | 16 |
| #10 | #1 OR #2 OR #3 OR #4 OR #5 OR #6 OR #7 OR #8 OR #9 | 44 |

**Supplementary Table 2**. Search strategy for Scopus

| **Search Set** | **Scopus** | **Results** |
| --- | --- | --- |
| #1 | (rs17782313 AND appetite) [TITLE-ABS-KEY] | 12 |
| #2 | (rs17782313 AND satiety) [TITLE-ABS-KEY] | 9 |
| #3 | (rs17782313 AND “energy intake”) [TITLE-ABS-KEY] | 16 |
| #4 | (rs17782313 AND “eating behaviour”) [TITLE-ABS-KEY] | 15 |
| #5 | (rs17782313 AND “eating behavior”) [TITLE-ABS-KEY] | 15 |
| #6 | (rs17782313 AND ghrelin) [TITLE-ABS-KEY] | 5 |
| #7 | (rs17782313 AND gluttony) [TITLE-ABS-KEY] | 0 |
| #8 | (rs17782313 AND hunger) [TITLE-ABS-KEY] | 5 |
| #9 | (rs17782313 AND leptin) [TITLE-ABS-KEY] | 19 |
| #10 | 1 OR 2 OR 3 OR 4 OR 5 OR 6 OR 7 OR 8 OR 9 | 49 |

TITLE-ABS-KEY: Title, abstract and key words

**Supplementary Table 3**. Search strategy for Web Of Science

| **Search Set** | **Web of Science** | **Results** |
| --- | --- | --- |
| #1 | (rs17782313 AND appetite) [All Fields] | 10 |
| #2 | (rs17782313 AND satiety) [All Fields] | 9 |
| #3 | (rs17782313 AND “energy intake”) [All Fields] | 22 |
| #4 | (rs17782313 AND “eating behaviour”) [All Fields] | 3 |
| #5 | (rs17782313 AND “eating behavior”) [All Fields] | 21 |
| #6 | (rs17782313 AND ghrelin) [All Fields] | 6 |
| #7 | (rs17782313 AND gluttony) [All Fields] | 0 |
| #8 | (rs17782313 AND hunger) [All Fields] | 7 |
| #9 | (rs17782313 AND leptin) [All Fields] | 19 |
| #10 | 1 OR 2 OR 3 OR 4 OR 5 OR 6 OR 7 OR 8 OR 9 | 58 |

**Supplementary Table 4**. Search strategy for Cochrane Library

| **Search Set** | **Cochrane** | **Results** |
| --- | --- | --- |
| #1 | (rs17782313 AND appetite) [All Fields] | 0 |
| #2 | (rs17782313 AND satiety) [All Fields] | 0 |
| #3 | (rs17782313 AND “energy intake”) [All Fields] | 0 |
| #4 | (rs17782313 AND “eating behaviour”) [All Fields] | 0 |
| #5 | (rs17782313 AND “eating behavior”) [All Fields] | 0 |
| #6 | (rs17782313 AND ghrelin) [All Fields] | 0 |
| #7 | (rs17782313 AND gluttony) [All Fields] | 0 |
| #8 | (rs17782313 AND hunger) [All Fields] | 0 |
| #9 | (rs17782313 AND leptin) [All Fields] | 0 |
| #10 | 1 OR 2 OR 3 OR 4 OR 5 OR 6 OR 7 OR 8 OR 9 | 0 |
